# Using Spike Gene Target Failure to Estimate Growth Rate of the Alpha and Omicron Variants of SARS-CoV-2

**DOI:** 10.1128/jcm.02573-21

**Published:** 2022-03-07

**Authors:** Benjamin F. Smith, Peter F. Graven, Doris Y. Yang, Siouxzanna M. Downs, Donna E. Hansel, Guang Fan, Xuan Qin

**Affiliations:** a Department of Pathology & Laboratory Medicine, Oregon Health & Science University, Portland, Oregon, USA; b Office of Advanced Analytics, Oregon Health & Science University, Portland, Oregon, USA; c School of Public Health, Oregon Health & Science University, Portland, Oregon, USA; Cepheid

**Keywords:** Alpha, case doubling time, SARS-CoV-2, SGTF, delta, omicron

## LETTER

The severe acute respiratory syndrome coronavirus 2 (SARS-CoV-2) pandemic has proceeded in multiple waves since late 2019, partly driven by the emergence of successive variants. Worldwide sequencing of the viral genome has yielded unprecedented real-time tracking of the evolution of SARS-CoV-2 (1). Alpha emerged in late 2020 (2) and was succeeded by Delta, which predominated in the latter portion of 2021. In late 2021, Omicron displaced Delta (3; https://covid.cdc.gov/covid-data-tracker/#variant-proportions).

Detection of SARS-CoV-2 infection using a PCR-based method is the gold standard for molecular diagnosis. At our institution, the TaqPath COVID-19 multiplex PCR assay (Thermo Fisher, USA) was deployed in October 2020 and became the primary clinical testing modality. It includes primers for three viral targets: N, ORF1AB, and S genes (4). Deletion of codons 69 and 70 in the S gene, common to Alpha (5) and Omicron (6), results in S gene target failure (SGTF). We used SGTF with N gene cycle threshold (*Ct*) < 30 as a proxy for lineage to monitor the early growth rate of Alpha and Omicron by calculating case doubling time. Lineage designation was later supported by viral genome sequencing via NextSeq (Illumina, USA), also performed on samples with N gene *Ct *< 30. For comparison, Delta case doubling time was calculated using sequencing-confirmed samples.

To estimate the case doubling time of the variants based on frequency of cases rather than count, a model was constructed to show growth relative to existing variants. Following the approach outlined by Volz et al. (7), in a model where strains are competing in the population and growth of existing strains is assumed to be constant at some level, R, prior to the introduction of the new strain, the variant can be assumed to have a reproduction number of R plus some percentage (1+s). In this model, the log odds of the variant share will be proportional to (R/g)st, where g is the generation time, s is a selection coefficient, and t is time. If a logistic model is fit with the shares of variant and time, the estimated coefficient on time is thus equal to (R/g)s. If R is assumed to be 1, reflecting stable growth conditions, and g is assumed to be 6.5 days, then b = (1/6.5)*s and s = b*6.5. This estimate of the growth rate per generation can be converted to doubling days using quotient of natural logs whereby the case doubling rate (in days) is equal to natural log (2)/natural log(s).

**FIG 1:**
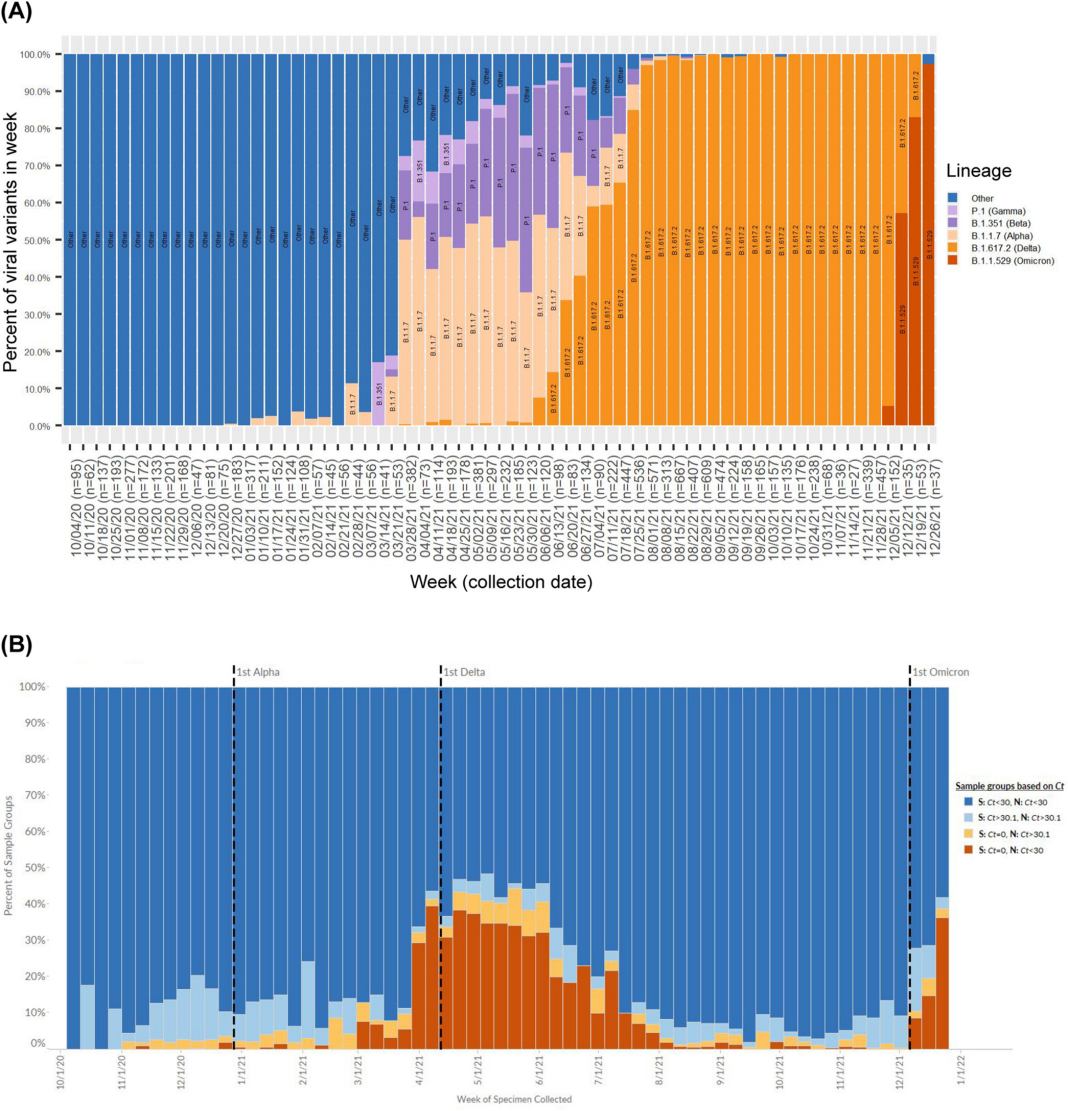
(A) At OHSU, 12,474 viruses were sequenced with collection dates between October 4, 2020 and December 29, 2021. A total of 1,415 sequences were identified as Alpha, collected between December 29, 2020 and August 30, 2021. There were 6,399 sequences identified as Delta, collected between April 15, 2021 and December 22, 2021. There were 108 sequences identified as Omicron, collected between December 8 and 29, 2021. (B) SGTF appeared concurrently with sequencing-confirmed samples of the Alpha and Omicron variants, with very low levels of SGTF reported in the period of Delta predominance (August through mid-December, 2021). SGTF is defined by samples with S *Ct *= 0 with N *Ct *< 30 (dark orange). Samples with S *Ct *= 0 and N *Ct *> 30.1 (light orange) or S *Ct *> 30.1 and N *Ct > *30.1 (light blue) were considered nonspecific. Samples with S *Ct *< 30 and N *Ct *< 30 (dark blue) were negative for SGTF.

At our institution, the first sequencing-confirmed Alpha was collected on December 29, 2020, the first Delta on April 15, 2021, and the first Omicron on December 8, 2021 (Fig. 1A), with SGTF appearing shortly after Alpha and Omicron (Fig. 1B). Alpha had a case doubling time of 9.54 days based on 6,931 samples with SGTF. Delta’s case doubling time was 19.30 days based on 447 sequenced samples. Omicron’s case doubling time is 4.28 days, nearly half that of Alpha, based on 1,078 samples with SGTF since December 8, 2021.

This shortened case doubling time is additional evidence of the increased transmissibility of Omicron. Using SGTF provides a real-time estimate of viral epidemiology for Omicron; however, there are limitations with this analysis. Rarely, SGTF can be detected in sublineages of the Delta variant that are simultaneously in circulation (8). Also, the time it took for Delta to come to predominance was complicated by several factors including remote schooling, increased outdoor activities during summer, the protection from vaccination in the population, and the simultaneous presence of Alpha, Beta, and Gamma variants in circulation. All of these may have contributed to Delta’s prolonged case doubling time compared with that of Alpha and Omicron. Nonetheless, clinical laboratory data are essential for monitoring the SARS-CoV-2 pandemic. The case doubling time in our community reflects the nationwide trend toward Omicron predominance and immune evasion, and can inform local pandemic mitigation efforts. In conjunction with early reports of the clinical severity of and immunologic responses to a given variant, case doubling time can be used to inform whether to implement a variety of public health measures, e.g., masking policies, hospital visitor policies, vaccination booster recommendations, scale-up of laboratory testing, and widespread distribution of at-home testing.
